# Effectiveness of a Novel Index System in Preventing Early Hearing Loss among Furniture Industry Skills Training Students in Malaysia

**DOI:** 10.3390/ijerph17218032

**Published:** 2020-10-31

**Authors:** Khairul Azhar Abdul Rahim, Jegalakshimi Jewaratnam, Che Rosmani Che Hassan, Mahar Diana Hamid

**Affiliations:** Department of Chemical Engineering, Faculty of Engineering, University of Malaya, Kuala Lumpur 50603, Malaysia; kva170072@siswa.um.edu.my (K.A.A.R.); rosmani@um.edu.my (C.R.C.H.); mahar.diana@um.edu.my (M.D.H.)

**Keywords:** Hearing Conservation Program (HCP), hearing conservation index system, noise exposure, compliance, management and monitoring tool

## Abstract

Occupational noise-induced hearing loss (ONIHL) is the most reported occupational disease in Malaysia. ONIHL is aggravated by the presence of early hearing loss amongst the youth prior to entering a real working environment. At technical and vocational education training (TVET) institutions, students may develop early ONIHL because training workshops are designed imitating the industrial working environment to produce skilled workers. The exceeding noise level at workshops and recent risk of non-occupational noise can cause early ONIHL among these students. Therefore, ONIHL must be addressed at the early stage of producing skilled workers. Octa hearing conservation index (OHCI) system is developed as a management and monitoring tool for hearing conservation program (HCP) in TVET institutions. Six existing and two new HCP components were used to build the index system. A pilot test on the effectiveness of the OHCI system was conducted in a selected TVET institution for six months. The post-HCP shows a 52.6% improvement compared to the pre-HCP. The implementation of HCP has shown improved awareness on the hazards of loud noise exposure and active use of hearing protection devices among participants. The OHCI system has a great potential as a tool to improve HCP implementation in TVET institutions, and eventually, industry.

## 1. Introduction

The World Health Organization (WHO) estimated that around 466 million people are suffering from hearing loss globally [1]. The prevalence of hearing loss is predicted to increase to 630 and 900 million people by 2030 and 2050, respectively. A common cause for hearing disorders is exposure to loud noise [2]. The expansion in the industrial sectors and demographic population shifts has caused exposure to harmful noise at the workplace increase exponentially. Hearing loss due to exposure in the workplace is termed as occupational noise-induced hearing loss (ONIHL). ONIHL is a serious issue because it has a huge impact on both individual health and the economy worldwide. In a review, Huddle et al. [3] elaborated that hearing loss causes billions of dollars lost worldwide. The losses come from lifetime disability cost of affected individuals, medical expenditures, loss of income and productivity, caregiver costs, and workers’ compensation payment. ONIHL has been there since the industrial revolution era. Though ONIHL can result in permanent hearing loss, it is a highly preventable disease. Preventive measures must be implemented to apprehend the severity. There is continuous effort worldwide to address this issue via technology, regulations, awareness programs, and research and development [4,5,6]. In a report on disability, the WHO and World Bank has categorized three tiers of preventive approach [7]. Primary prevention aims to prevent adverse health conditions at the earliest stage. In this stage, a preventive approach, such as conducting awareness programs, is executed to act as an early intervention to avoid adverse health conditions. Secondary prevention aims for early detection of health problems and treatment. Tertiary prevention focuses on controlling and reducing the impact of health problems and promotes recovery.

In Malaysia, ONIHL is the most reported occupational disease every year. In 2019, more than 90% of the occupational disease cases reported was ONIHL [8], and this has also been the trend in the past years [9]. Manufacturing industries have the noisiest working environment. Metal industries recorded the highest percentage of exposure to hazardous noise levels and prevalence of hearing loss and impairment to the workers [10]. Activities, such as grinding, use of power tools, and heavy machinery, had exposed the workers to harmful doses of noise [11,12,13]. The risk is greater when performing welding activities where workers are exposed to the combining effect of ultrasonic and loud audible noises [14]. The second noisiest industry is the furniture industry [15,16,17]. Most of the activities in wood product processing, were found to emit harmful levels of noise [12,17]. 

Tahir et al. [15] studied occupational noise exposure in 26 manufacturing industries in Malaysia and found that 72% of the industries were exposed to a noise level of 86–90 dBA. In 2014, when the study was conducted, 90 dBA was the permissible exposure limit (PEL). In 2019, the new PEL was reduced to 85 dBA with an action level of 82 dBA and an exchange rate of 3 dBA [18]. With the new PEL, more industries may record non-compliance. Even the 85 dBA PEL is still considered high by some researchers. The 85 dBA is based on a time-weighted average (TWA) of 8 h. Shift working hours in the industry is 12 h, and high decibel exposure can occur outside working hours too [19]. There are studies proposing PEL below 70 dBA [20,21]. This level happens to be more suitable by taking into consideration of total daily noise dose. Furthermore, life-time noise exposure needs to be adjusted to suit the aging population demographic shift. The lower PEL is definitely favorable to prevent ONIHL. However, the implementation may require a high cost of engineering control. The present industrial equipment may require major modification.

As a preventive approach, an existing hearing conservation program (HCP) was adopted by the Department of Occupational Safety and Health (DOSH), Malaysia, to manage and control the risk arising from occupational noise exposure [22]. HCP is a structured program designed to reduce, minimize, and eliminate the risk of excessive noise exposure to the affected workers. Researchers insist that the implementation of HCP workplaces is the best remedy to apprehend ONIHL [15,23,24]. The effectiveness of HCP in preventing hearing disorder among workers can be determined through the evaluation of completeness, quality of the program components, and assessment of the audiometric testing results [25,26,27]. The implementation of HCP may vary worldwide, but the evaluation components used are quite common. The seven common components are noise exposure monitoring, noise control, provision of Hearing Protection Device (HPD), audiometric testing, education, and training program, record-keeping, and program evaluation [22,27,28]. Australia and Singapore adopted these HCP elements [29,30]. In Malaysia, HCP was introduced through the amendment of noise regulation 1989 [31] and enhanced through the newly gazetted noise regulation in 2019 [18]. In the newly published industrial code of practice, the HCP in Malaysia adopted all of the common components [22]. The implementation of the new regulation has been widened to almost all workplaces that expose workers to hazardous noise levels. Since HCP has been gazetted legally, employers are obliged to implement comprehensive HCP. However, the level of awareness and implementation of control measures were found to be low in these industries [32,33]. 

Small and medium industries (SMI) employers dutifully provide HPDs, but neglect other components of HCP, especially noise control measures [32,33,34]. The employers are unaware that the effectiveness of one size to fit everyone HPD in protecting users against harmful noise remains questionable. In order to ensure sufficient protection, HPD fitting must be done on an individual basis. Individual fitting allows users to select ergonomically comfortable and adequately attenuating HPD [35]. The most common method used to estimate the adequacy of hearing protector attenuation is a noise reduction rating (NRR) [36]. NRR is a single-number index used to measure sound attenuation. Even with appropriate HPD selection, optimal protection depends on the wearers’ attitude and ability to fully fit the HPD in ear canals. Furthermore, a recent study debates that NRR can be an unreliable value because NRR is developed in laboratories without actual workplace noise [37]. Therefore, it is critical to assess the actual attenuation through individual HPD fit testing on-site [38]. Individual fit testing can provide information to users on whether the HPD is being worn correctly to give sufficient protection. In developing holistic HCP, the effective fit testing element must be included. The availability of HPD field fit-test methods, such as subjective real-ear attenuation, at threshold (REAT) measurements, and objective field microphone-in-real-ear (F-MIRE) measures should be used to ensure effective HCP implementation [39]. A successful implementation of HCP will certainly contribute to controlling occupational noise by reducing the exposed dose and increasing the use of HPD [40]. Arezes and Miguel [41] found that individual risk perceptions and HCP were direct factors that influenced the use of HPD among workers. For the workers already affected by ONIHL, the implementation of HCP and HPD use can help in preventing the worsening of hearing loss [24].

Previous best practices revealed that two elements play a significant role in ensuring successful HCP management and monitoring. The elements are a strong management commitment delivered via policies [38,42] and the appointment of a key individual as an implementer or hearing conservation administrator (HCA) [22,26]. It is essential to include these two elements into Malaysia’s HCP components list. In addition, a user-friendly HCP management system can ease smooth execution at workplaces [43]. Since the effectiveness of HCP implementation must be assessed regularly, manual auditing using pages of the checklist can be time-consuming and less attractive to the employers. The audit reports need to be archived manually to track compliance improvement for a progressing time period. In the event of staff mobilization, the documents tracking can be tedious and go missing as well. Furthermore, the heuristic method of self-reporting assessment can be biased and inconsistent [40,44]. A simple rubric system that is quick and easy in auditing and archiving HCP compliance of a premise at a particular time period could encourage more employers to commit to HCP management and monitoring. The use of interactive and innovative technology can improve the effectiveness of HCP implementation and ensure none of the components are comfortably ignored [45]. A system that provides a visual compliance summary can easily aid management in identifying components that need improvement. A comprehensive and user-friendly hearing conservation program can be developed and implemented to inculcate good practices in preventing ONIHL.

With the multiple harmful effects of occupational noise toward human and national economic growth, the issue of occupational noise hazards must be addressed at the fundamental stage of producing young, skilled workers. The focus of this paper is primary prevention. In the past, hearing loss used to be commonly associated with the aging population. In the millennia, hearing loss occurs at an earlier age, due to occupational or recreational noise exposure [1,2]. The increase in non-occupational hearing loss among the youth is worrisome. In the United States of America, 19% of young adults aged 20 to 29 were experiencing a hearing threshold shift [46]. Early exposure to loud noise has the potential to develop premature hearing loss and will become more severe when these young people start working in the industry with exposure to occupational noise hazard. Therefore, intervention programs are being implemented for young people to address premature hearing loss. One of the famous hearing loss intervention programs for young people is the Dangerous Decibels introduced in the United States of America (USA) [47]. This program has been implemented worldwide in various school intervention programs aims to increase awareness of noise exposure among students. A governmental preventive campaign called ‘Iets Minder is de Max’ has been initiated in Belgium aims to prevent ONIHL among adolescents [48]. Another successful program called Agricultural Disability Awareness and Risk Education (AgDARE) was conducted in the United States of America (USA) to increase safety and health practice and awareness among adolescent farm children [49,50]. The online resources like the “it’s a noisy planet” website [51] and the New York city department of environmental protection website [52] can be useful sources for educational materials. These online sources provide education modules that can be used by educators as additional curricula. Available interactive materials can be applied for classroom activities. Beside the initiatives outlined above, educators can take advantage of the open-access online resources of hearing loss education material available worldwide [53].

In Malaysia, there is no evidence that shows such programs being implemented in any educational institution, including technical and vocational education training (TVET) institutes. In the Eleventh Malaysia Plan 2016–2020, TVET institutes are expected to produce 35% of domestic skilled workers by 2020 [54]. TVET institutions are focused on the young workforce developing skills that can be used in various industries. Therefore, TVET students are exposed to similar hazards as in the industries because they are trained using industrial-scale machinery and equipment. With the emergence of recreational and non-occupational noise exposure, TVET students pose a greater risk of developing early effects of ONIHL. An intervention program must be introduced to them during their study duration as a preventive measure. The young TVET graduates, fed with ample knowledge and awareness, will certainly contribute to the positive HCP compliance in the industry.

In this work, a novel octa hearing conservation index (OHCI) system is developed as a tool to manage, monitor, and evaluate the implementation of HCP in a TVET institute. Furniture manufacturing is known to have a noisy working environment and among the main contributor to ONIHL cases reported worldwide [17,55,56]. The public TVET institutes in Malaysia are designed to provide practical training to students in a similar working environment as in the real industry. About 70% of the learning activities are conducted through practical training, which means students will spend most of their time in the workshop. The students and lecturers are using real industrial machinery, thus, making them vulnerable to similar hazards as in the industry [57,58,59]. Therefore, a TVET institute teaching furniture manufacturing course was selected to test the OHCI index system.

This OHCI system incorporates eight HCP components. Six components that are noise exposure monitoring, noise control, provision of hearing protection device (HPD), audiometric testing, education, and training program; and record-keeping was adapted from the existing HCP in Malaysia. Two new components (policy and the HCP team) were added in this study. Each HCP component is assessed against the compliance indicator developed from the legal requirements. A total of 40 indicators for 8 HCP components was assessed through conformity assessment before and after HCP implementation. OHCI is calculated for each HCP component. An octagon visual chart is plotted to summarize the compliance for the pre- and post-HCP. The HCP compliance was then classified into OHCI class. HPD usage was observed to evaluate participants’ attitudes and practices. Personal noise exposure, area noise monitoring, and self-reporting knowledge and ability were measured to study the effectiveness of proposed HCP implementation. 

## 2. Materials and Methods 

### 2.1. HCP Components and Conformity Assessment

In ensuring the effectiveness of HCP to prevent ONIHL, all components must be collectively implemented [25,26,32]. The existing HCP has six components, as outlined in the noise regulation [18] and the practical standard of the Industrial Code of Practice (ICOP) [22]. The six HCP components are noise control, noise monitoring, HPD, audiometric testing, education and training, and record-keeping. In this study, two more components, which are policy and HCP team, were introduced into HCP. In total, the HCP proposed in this study has eight components. Conformity assessment is used to assess the level of compliance for each component. The assessment consists of indicators outlined in the occupational noise exposure regulation [18], the practical standard of ICOP [22], and best practice approaches from previous studies [60]. The indicators for each HCP component are shown in Table 1. A total of 40 indicators was set for eight HCP components. The level of compliance for each component was assessed by evaluating the respective indicator and assigned score. A three weighted scoring system was used to evaluate conformant against each indicator, where zero marks for non-conformant, one mark for partially conformant, and two marks for fully conformant. The score for each indicator was added and divided by the total full score of each component to obtain a mean component score. The total full score of each component is the summation of fully conformant scores for all indicators in an HCP component. The mean component score was converted to component compliance percentage. The compliance percentage was assigned to an OHCI score, as shown in Table 2.

### 2.2. Concept of OHCI

**Figure 1:**
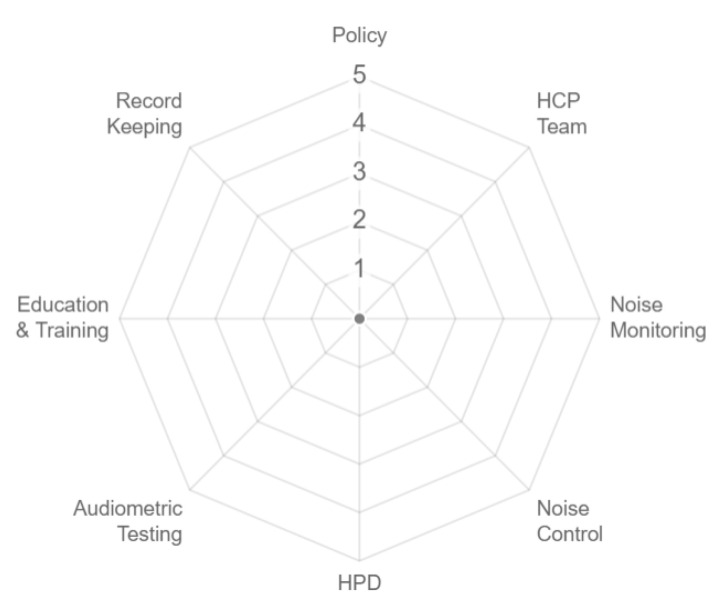
Octa-Hearing Conservation Index (OHCI) system.

The OHCI score for each HCP component was plotted into the OHCI system. A novel OHCI system was developed in this study to deliver a graphical representation of the HCP compliance level. Figure 1 shows the concept of the OHCI system. The five scales in the OHCI system indicate the level of compliance for each component. The overall HCP compliance level is determined based on the octagon shape and mean index. If all components are fully compliant, a complete symmetrical octagon shape will be obtained. If the HCP is partially complied, an uneven shape will be formed. At a glance, the octagon shape gives an idea of areas that needs improvement. Progressive improvement in the HCP can be easily seen through the improving octagon shape. The OHCI score of each component was used to calculate the mean OHCI. The mean OHCI is assigned into five classes, as shown in Table 2. The classes give a numerical rating to the different shapes of the octagon. 

### 2.3. Field Work and Data Collection

A TVET institute located in Kuala Langat district, Selangor state in Malaysia, which provides furniture manufacturing technology courses, was chosen as a sampling location to validate the OHCI index system. Upon obtaining permission from the institute director, the study was conducted for six months. The duration of six months was chosen, as suggested by Sayapathi, Su, and Koh [61], as a minimum interval to sustain the awareness level on occupational noise exposure. There were 47 students and five lecturers in the institute who were the participants of this study. The participants never had any hearing problem, did audiometric testing, or attended any formal education program on hearing conservation.

This study requires pre- and post-HCP data. The initial conformant assessment was conducted in July 2019. The compliance level for each HCP components was assessed through work process monitoring and evidence-based audit. The pre-HCP OHCI was presented to the top management of the institute. The recommendations for improving HCP compliance from the discussion were transferred to the HCP team through consultation sessions. The HCP team was instructed to conduct a two days training program on HCP and noise exposure in the first month of this study for the participants. The training materials were prepared by the researcher. An awareness program on HPD was conducted for the HCP team, and participants. Disposable HPD with Noise Reduction Rating (NRR) of 29 dB was supplied for the participants’ use. The HPD usage after the awareness program was monitored daily. 

After six months, post-HCP data collection was conducted in January 2020. The pre- and post-HCP OHCI scores were compared to determine the effectiveness of HCP implementation. A paired-samples *t*-test was conducted to evaluate the statistical significance of OHCI scores differences. Results with *p* < 0.05 indicate a significant statistical difference.

A self-reporting knowledge and abilities assessment survey was conducted to study the effectiveness of HCP activities in improving participants’ awareness of HCP and noise exposure. A set of questionnaires consisting of 15 close-ended questions covering the general aspects of hearing loss, noise exposure, HCP, and HPD was used to evaluate participants’ level of knowledge and abilities. The questionnaire shows acceptable reliability with Cronbach’s alpha α = 0.85. Participants were invited to join the survey on a voluntary basis. 33 out of 47 participants attended both the pre- and post-HCP self-reporting knowledge and abilities surveys. This represents 70.2% of the students’ population. 14 (29.8%) responses were rejected because the students only participated in either pre- or post-HCP survey sessions. From the 33 students, 54.5% (*n* = 18) were female and 45.5% were male students. The mean age of the students is 19.6 (SD = 0.6). Both the pre- and post-HCP results were analyzed using IBM SPSS Statistics for Windows version 25 (IBM Corporation, Armonk, New York, NY, USA). 

Area noise emission monitoring was conducted at the institute’s workshop. Students spend at least 8 h a day during class activity in the workshop. Sound Level Meter (SLM) CPS SM150 (CPS, Miramar, Florida, FL, USA) was used to record the noise emitted by each activity, machine, and tool used in the workshop. The SLM and its accessories, such as microphone and associated cables, were chosen to meet the requirements for IEC 61672-1:2002, Class 2 instrumentation.

**Figure 2:**
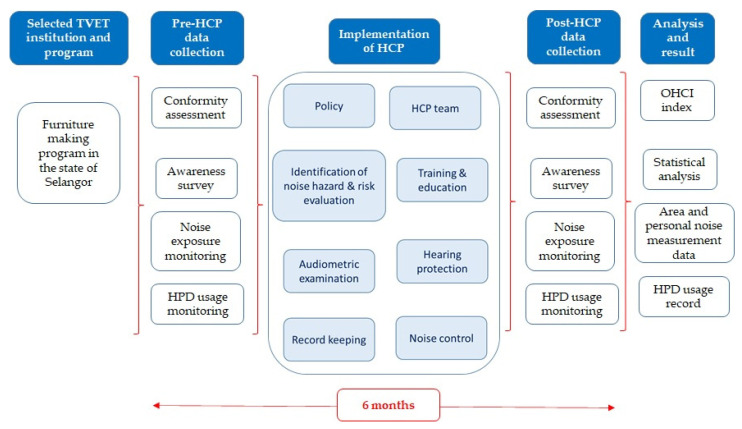
Study framework.

Personal noise exposure monitoring was also conducted as per procedures outlined by the international standard of ISO 9612: 2009 Acoustics-Determination of occupational noise Exposure-Engineering method [62]. Three voluntary participants were selected to undergo personal noise exposure monitoring. Noise dosimeters were attached to the selected participants for 8 h during their class activity in the workshop. 3M Edge, e.g., four personal dosimeter (3M-Quest Technology, Oconomowoc, WI, USA) with an exchange rate setting of 3 dB was used. The full-day noise exposure level obtained was compared to the newly regulated PEL of 85 dBA and an action level of 82 dBA with an exchange rate of 3 dBA [18]. Both the dosimeter and sound level meter were calibrated before and after each measurement. The pre- and post-HCP data- were analyzed using 3M Detection Management Software (3M DMS) (3M-Quest Technology, Oconomowoc, WI, USA). A summary of the study framework is given in Figure 2, below.

## 3. Results and Discussion

### 3.1. HCP Components Conformity Assessment and OHCI System

Table 3 shows the pre- and post-HCP conformity assessment results. Overall, the pre-HCP conformity assessment shows a poor level of compliance. The OHCI scores were either 0 or 1. The poor compliance in pre-HCP suggests that HCP was never implemented in the TVET institution. A discussion with the top management on the outcome of pre-HCP revealed that the management was not aware of the obligation to conduct HCP in the institution. Little to no awareness and knowledge on noise regulation and HCP among the management and educators in the institution significantly affects the HCP compliance [34,63].

The management admitted that some of the preventive measures were implemented to comply with general safety regulations and not noise regulation specifically. HPDs were provided because it is a very basic and common personal protective equipment (PPE). The HPDs were procured without considering the noise reduction rating (NRR) and fit-test. In education and training activities, workplace noise hazard was introduced superficially without paying much attention to important topics, such as noise permissible exposure level and precautionary steps. Due to the unavailability of specific noise, related policy in the TVET institute, noise monitoring and audiometric testing, were never done. There was also no record of HCP in the workplace.

After the pre-HCP result discussion, the top management has agreed to establish HCP at the institution. A policy was developed to incorporate HCP in the training operation. The HCA and HCP teams were assigned to conduct HCP. The pre-HCP results were used as a baseline reference to planning the actions for each HCP components. Interventions, such as enforcing the use of HPD, warning signage, and conducting educational, and a training program was organized for educators and students.

After six months of HCP implementation, post-HCP conformity assessment shows a significant increase in the level of compliance for all components. Four components, namely, noise control, HPD, education and training, and record-keeping, reported full compliance. Policy and noise monitoring components had an index score of 4. The policy component missed full compliance, due to improper planning for audiometric test and noise monitoring. This is attributed to budget constrains. HCP implementation in the institution only began in July 2019, but the budget planning is usually done at the beginning of the year. Partial compliance was obtained for audiometric testing because without budget allocation; audiometric test cannot be done properly. The HCP team component also showed partial compliance because all the team members were not sent for any competency training on HCP and noise exposure. It is essential for the top management to allocate sufficient budget to conduct HCP activities to achieve full compliances for all the components [64]. The policy and HCP component introduced in this study, proved effective. The establishment of a policy and HCP team ensured the smooth execution of each component as planned [65]. The score differences were statistically significant for policy, noise monitoring, HPD, education and training, and record-keeping. There is no significant difference statistically for the HCP team, noise control, and audiometric testing. 

**Figure 3:**
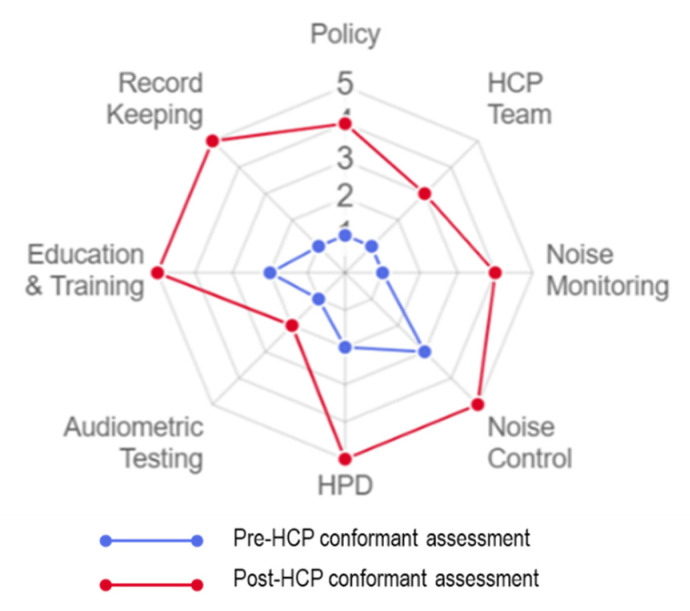
Visual representation of pre- and post-OHCI system.

The OHCI score for each component was plotted on the OHCI system to produce an octagon chart. Figure 3 show the pre- and post-OHCI visual representation. The overall pre-HCP OHCI shows an unhealthy octagon shape. The mean OHCI for the plot is 1.5 (SD = 0.8) and classified into OHCI class 2, which indicates poor to fair compliance.

The overall post-HCP shows an improved octagon shape, though not perfect. The mean OHCI for the plot is 4.1 (SD = 1.1), and classified into OHCI class 5, indicating good to excellent compliance. The OHCI, mean OHCI, and OHCI class improvement were proved statistically significant with *p* < 0.001.

The OHCI system provides useful and quick information for looking at the octagon shape components which need improvement. Best practices should be continued can be easily identified without having to glance through several tables and calculations. This visual feature is particularly of interest to the management because the effectiveness of HCP implementation can be assessed and understood easily. The OHCI system can definitely be used as an HCP monitoring tool in educational institutions and industry workplaces. The system can also serve as compliance evidence for regulatory body auditors.

### 3.2. HPD Usage Report

**Figure 4:**
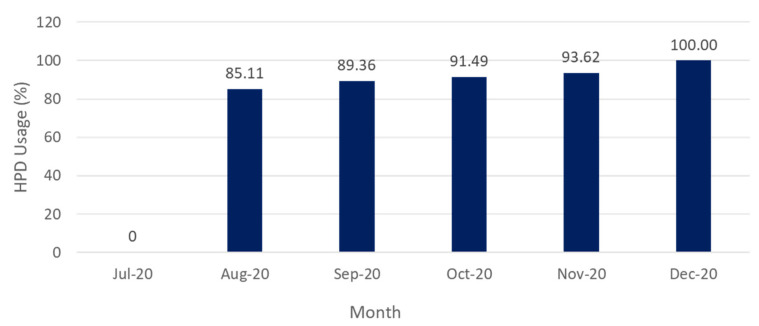
The trend of HPD usage among participants.

The percentage of HPD usage is determined based on the number of activities and HPD users. In July 2019, during pre-HCP, there is no record of HPD usage by the students during their class activities in the workshop. Though the workshop environment is found to be noisy, the usage of HPD were low, just as reported by NS and M [58]. From August to December 2019, HPD usage shows an increasing trend, as illustrated in Figure 4. After the pre-HCP assessment, the activities required in HPD component indicators were implemented. HPD training session that involves module contents, such as HPD fitting procedure, NRR calculation, and HPD maintenance, has helped students better understand the importance of HPD. The HPD usage hit 100% in December 2019, which was the final examination month. During the final exams, it is the institute’s practice that students must wear appropriate personal protective equipment (PPE) to gain access to the examination area. With the HPD knowledge from training, all the students included disposable earplugs in their PPE. Sufficient HPD provision, rules, and regulation enforcement has encouraged HPD usage among students. Similar trends were also reported by Trabeau et al. [65], Thepaksorn et al. [66], and Beach, Nielsen, and Gilliver [67] post-HPD training.

It is a concern if the HPD used in this study provided sufficient sound attenuation for optimal protection to all the participants. During HPD training, the participants were given exposure to the general concept of the fitting test, HPD selection based on NRR ratings, and correct method to wear HPD. However, the participants were provided with the same type of HPD in this study. Therefore, the HPDs may not be ergonomically suitable for everyone. The exact individual attenuation cannot be identified, due to the unavailability of individual fit-testing data. Similar to the audiometric test, the TVET institution could not invest in technology-based HPD fit-testing, due to budget constrains. HPD fit-testing must be included in the HCP planning to ensure an effective HCP outcome. TVET institutes should invest in technology-based products, such as real-ear attenuation at threshold (REAT) and field microphone-in-real-ear (F-MIRE) [39]. These products can be valuable training tools for TVET students. Personal attenuation rating (PAR) from fit-testing measurement can show the actual noise attenuation provided by the selected HPD. Individual fit-testing can definitely increase awareness among users on the importance of using correct HPD, and subsequently, contribute toward holistic hearing loss intervention. These tools can boost the HPD component compliance in HCP.

### 3.3. Improvement in Self-Reporting Knowledge and Abilities

Table 4 shows the result of self-reporting knowledge and abilities survey. The pre-HCP result shows a poor level of self-reporting knowledge and ability with a mean score of 2.4 (SD = 0.4). The students have little knowledge of occupational noise hazards and awareness on hearing protection practice. This outcome matched a similar study conducted by DelGiacco and Serpanos [68].

After six months of HCP implementation, the same participants reported an increase in knowledge and awareness about the same subjects with a mean score of 3.3 (SD = 0.3). A paired-samples *t*-test revealed that 13 out of the 15 items show statistically significant difference. There is no statistically significant difference between pre- or post-HCP score for item 7 and 11.

During the HCP implementation, all students were required to attend two days of training session on noise hazards at the workplace. Training modules included information on the hearing mechanism, effects of hearing loss, noise measurement, and proper use and maintenance of HPD. This session has helped students gain knowledge and improved their work practice, similar to findings by Reddy et al. [69]. A practical session, such as recording noise from their work activities and measuring noise emitted from machinery, has provided further understanding of the risk of noise to their hearing ability. Comprehensive training must be conducted regularly to increase the awareness level among the students [61,70]. HCP team can adapt the available online resources to improve the educational and training activities [51,52,53].

### 3.4. Noise Exposure Monitoring and Assessment of Proposed Control Action 

Table 5 shows the source measurements and personal noise exposure recorded during the noise exposure monitoring activities. The pre-HCP area noise exposure monitoring recorded five work activities emitting hazardous noise level, which is beyond 85 dB in the furniture technology workshop. The highest noise level recorded is from the surface planning work with a mean noise level of 101.1 (1.3) dBA. Other training activities that emitted high noise level was wood cutting, wood shaping, wood edging and parts assembly. Based on the observation, a high sound pressure level was contributed by the use of industrial-type machinery and electrical hand tools, such as a planer, router, sander, and multiple types of saw. These results are in agreement with Durcan and Burdurlu [16] and the machinery database produced by 3M [71]. The post-HCP area noise exposure monitoring did not obtain any significant different reading as there was no engineering control implemented into the workshop surrounding and machines. Budget constrains has refrained any engineering control to be implemented in the current year. As a substitution, administrative control was used by implementing a suitable timetable or shift system for the class activities conducted in noisy areas of the workshop to reduce exposure duration.

The mean noise exposure level recorded pre-HCP was LAeq = 90.7 (2.4) dBA. Post-HCP data collection recorded lower exposure level of LAeq = 85.6 (0.6) dBA. There is a statistically significant decrease in personal noise exposure. At both times, participants are exposed to noise above the action level of 82 dBA the present workshop condition is nowhere near the 70 dBA proposed by Fink 2019 [21]. However, the implementation of administrative control to make a shift system for class activity in the workshop reduced the period and amount of exposure. With no engineering control available and the use of the same old and noisy machinery, the workshop still exposed students and staff to hazardous noise levels.

## 4. Conclusions

In this study, an HCP containing eight components were tested at a selected TVET institute. The aim of HCP is to increase ONIHL awareness and instill good hearing protection practices among students. The pre- and post-HCP conformant assessment showed significant improvement in compliance level within six months. The two new components, policy, and HCP team proved to be an essential addition. An appropriate policy to incorporate HCP in educational setting aid the success of the proposed HCP. Educational institution policymakers should adopt the latest technologies and explore interactive innovations to make HCP attractive to students. The HCP team plays a significant role in executing the program. The top management of TVET institutions should invest in HCP competency certification to better equip the team with knowledge and new information. The HPD usage increased significantly proving an increase in awareness level among the students post-HCP. The proposed HCP in this study can be improved by incorporating critical indicators, such as HPD fit-testing and audiometric testing. A self-reporting knowledge and ability survey post-HCP reveal that students were more knowledgeable about ONIHL and hearing loss prevention. The area noise emission and personal noise exposure were still above the PEL post-HCP. The institutions should consider engineering control measures when purchasing new equipment in the future to improve emission and exposure level, possibly reducing to below 70 dBA.

A novel OHCI system was developed and used to evaluate the effectiveness of HCP implementation using numerical and visual features. Each HCP component compliances were given OHCI scores. The OHCI scores were plotted into the OHCI system. The system produced a visual compliance chart. The chart was classified into OHCI class. The OHCI system was easy to use and provided quick information on the pre- and post-HCP compliance outcome to the management team. This pilot study shows that the OHCI system covers all the relevant parts required for HCP and is a reliable tool. The OHCI system can be used as effective HCP management and monitoring tool to comply with legal requirements. Employers can use OHCI as a justification tool for HCP budget planning. This system can also be used as supporting evidence by enforcers to determine the level of HCP compliance. Further multi-industry testing must be conducted to validate and enhance the effectiveness of the OHCI system.

## Figures and Tables

**Table 1:** Compliance indicators for each hearing conservation program (HCP) component.

Components	Policy (*n* = 5)	Noise Monitoring (*n* = 8)	Noise Control (*n* = 4)	Hearing Protection Devices (HPD) (*n* = 6)	Audiometric Test (*n* = 4)	Education and Training (*n* = 5)	Record-Keeping (*n* = 4)	HCP Team (*n* = 4)
**Indicators**	HCP Policy	Conduct identification of excessive noise annually	Implement engineering control solely or	Provision of HPD	Perform Audiometric testing on the exposed employee	Dissemination of information regarding noise exposure and HCP	Maintain a proper record of Noise identification assessment	Appointment of Hearing Conservation Administrator (HCA)
	Action plan for each HCP component	Conduct noise risk assessment by a registered assessor	Implement engineering control and administrative control or	HPD according to the approved standard	Notify employee within 21 days after report received	Dissemination of Instruction regarding noise exposure and HCP	Maintain a proper record of Noise risk assessment	HCA shall coordinate all aspects of HCP
	Establish target to reduce personal noise exposure level to below LAeq 82 dBA	Conduct Personal noise exposure monitoring	Implement administrative control solely	Provides full information on the attenuation values to employees and HCA	If negative for any STS inform the employee to maintain and improve preventive practice or	Organize training and education program regarding noise exposure	Maintain a proper record of Employee audiometric testing	HCA and team member shall possess knowledge on HCP and related legal requirement
	Review HCP annually	Conduct area noise exposure monitoring	Provision of HPD	HPD suitable to the working environment	Implement control measure for the employee with positive for TSTS and retest after three months or	Training on HPD	Maintain a proper record of Supporting documents on HCP implementation	HCA and team member shall be sent to HCP competency training
	Establishment of buy quiet policy	Use approved equipment for noise exposure monitoring	Designate hearing protection zone	Perform individual fitting test	Implement control measure for the employee diagnosed with ONIHL, HI, PSTS	Conduct awareness survey/ability assessment		
		Construct noise mapping	Enforce rules and regulation for the hearing protection zone	Consideration on the safety of the HPD wearer and others surround	Carry out audiometric test within three months after employee commencing work at noisy area			
		Findings and recommendation is presented to the exposed employee						
		Carry out report recommendation						

TSTS = Temporary standard threshold shift, HI = Hearing impairment, PSTS = Permanent standard threshold shift; n = Number of indicators.

**Table 2:** Octa hearing conservation index (OHCI) score, mean score, and class for HCP components compliance.

Compliance Level	Compliance Percentage	OHCI Score	Mean OHCI Score	OHCI Class
Excellent	80%–100%	5	4.01–5.00	5
Good	60%–79%	4	3.01–4.00	4
Moderate	40%–59%	3	2.01–3.00	3
Fair	20%–39%	2	1.01–2.00	2
Poor	0–19%	1	≤1	1

**Table 3:** Result of pre-HCP and post-HCP OHCI score.

HCP Components	Pre-HCP		Post-HCP		*p* Value
	Compliance (%)	OHCI Score	Compliance (%)	OHCI Score	
Policy	0	1	70	4	0.005
Noise monitoring	0	1	68.8	4	0.001
Noise control	50	3	87.5	5	0.058
HPD	25	2	100	5	0.001
Audiometric testing	0	1	37.5	2	0.058
Education and training	20	2	100	5	0.003
HCP team	0	1	50	3	0.092
Record-keeping	0	1	100	5	0.006
OHCI mean score	1.5		4.1		
OHCI class	2		5		

**Table 4:** Result of participants self-reported knowledge and ability.

Item	Subject	Pre-HCP (*n* = 33)		Post-HCP (*n* = 33)			Paired Samples *t*-Test	
		Mean	SD	Mean	SD	t	df	*p*
1	Knowledge on Occupational Noise Induced Hearing Loss (ONIHL)	2.5	1.1	3.6	0.94	−8.2	32	<0.001
2	Knowledge on social disadvantages of Occupational Noise Induced Hearing Loss (ONIHL)	3.1	1.1	3.7	0.8	−3.9	32	0.001
3	Knowledge on hearing mechanism	1.9	1.0	3.2	0.8	−7.0	32	<0.001
4	Knowledge on risk factors lead to hearing loss	2.7	0.9	3.3	0.7	−4.9	32	<0.001
5	Knowledge on purpose of audiometric testing	2.7	1.0	3.0	0.8	−2.2	32	0.037
6	Knowledge on noise control plan at the workplace	2.4	1.0	3.3	0.7	−4.5	32	<0.001
7	Ability to identify excessive noise warning sign at the workplace	2.8	1.0	3.2	0.7	−2.0	32	>0.05
8	Knowledge on rules and regulations in hearing protection zone	1.9	0.9	3.3	0.5	−8.9	32	<0.001
9	Ability to identify excessive noise source or area at the workplace	2.3	0.8	3.3	0.5	−8.1	32	<0.001
10	Knowledge on noise exposure limit	1.9	0.9	3.0	0.8	−6.0	32	<0.001
11	Knowledge on hearing conservation program (HCP)	2.6	0.8	2.9	0.9	−1.8	32	>0.05
12	Knowledge on the purpose of Hearing Protection Devices (HPD)	1.9	0.9	3.7	0.6	−8.8	32	<0.001
13	Ability to select appropriate type of HPD based on job	2.5	1.1	3.2	0.7	−5.0	32	<0.001
14	Ability to ensure HPD is properly fit when use	2.2	1.1	3.9	0.7	−8.9	32	<0.001
15	Ability to maintain good condition of HPD	2.2	1.1	3.3	0.7	−6.0	32	<0.001

**Table 5:** Noise exposure monitoring data.

Furniture Making Training Activities	Machines/Tools	Average Time (Min)	Pre-HCP Measurement		Post-HCP Measurement	
			Source Measurement (dBA) *n* = 3 Mean (SD)	Personal Exposure Measurement (dBA) *n* = 3 Mean (SD) LAeq	Source Measurement (dBA) *n* = 3 Mean (SD)	Personal Exposure Measurement (dBA) *n* = 3 Mean (SD) LAeq
Surface planing	Thicknesser planer Portable thicknesser planer	120	101.1 (1.3)	90.7 (2.4) *	100.7 (2.6)	85.6 (0.6) *
Wood shaping	Lathe machine Band saw Portable jig saw Scroll saw Orbital sander	120	96.2 (1.3)		95.8 (1.8)	
Wood cutting	Portable circular saw Sliding table saw Auto rip saw	90	98.7 (1.4)		98.9 (0.7)	
Wood edging	Portable trimmer Portable router Sanding machine Jointer planer	60	94.4 (0.8)		93.9 (1.2)	
Part assemble	Drilling machine Cordless drill	90	88.4 (1.6)		87.3 (0.8)	

* full training day (8.00 am–5.00 pm).
